# Associations of Sleep Quality and Physical Activity with Diabetes Quality of Life in Korean Americans with Type 2 Diabetes: A Cross-Sectional Study

**DOI:** 10.3390/healthcare12070756

**Published:** 2024-03-30

**Authors:** Mihyun Jeong

**Affiliations:** Department of Nursing, Gwangju University, Gwangju 61743, Republic of Korea; mjeong@gwangju.ac.kr

**Keywords:** diabetes quality of life, sleep quality, physical activity, Korean Americans, type 2 diabetes

## Abstract

The cross-sectional study aimed to examine the associations of sleep quality and physical activity with diabetes quality of life in Korean Americans with type 2 diabetes. A total of 119 Korean American adults with type 2 diabetes were recruited from Korean communities in Arizona, USA. The Pittsburgh Sleep Quality Index for sleep quality, the International Physical Activity Questionnaire for physical activity, and the Diabetes Quality of Life (DQOL) for quality of life were assessed. Descriptive statistics, bivariate correlations, and multiple linear regressions were conducted. The mean score of the total DQOL was 1.85 (SD = 0.28). Approximately 70% of those had poor sleep quality and a third showed low physical activity. The findings demonstrated that both sleep quality and low physical activity were independent predictors of overall DQOL scores, indicating that poor sleep quality and low physical activity are associated with reduced Diabetes Quality of Life in this demographic. Specifically, the satisfaction subscale of DQOL showed significant associations with low physical activity levels, suggesting that enhancing physical activity could potentially improve satisfaction within DQOL. Furthermore, the impact subscale was positively related to sleep quality, suggesting that better sleep quality might significantly lower the perceived negative impact of diabetes on daily life. No significant associations were found between sleep quality, physical activity, and the worry subscale of DQOL in the adjusted models. The study indicates that addressing sleep quality and promoting physical activity are essential components of diabetes management strategies aimed at improving the quality of life for Korean Americans with type 2 diabetes. This underlines the need for tailored interventions that consider cultural preferences and individual needs to enhance diabetes outcomes and quality of life in this population.

## 1. Introduction

Type 2 diabetes mellitus (T2DM) is a global public health challenge, affecting millions and resulting in significant morbidity, mortality, and economic burden [1,2]. The prevalence of T2DM has been increasing sharply over the last decades, accounting for more than 90% of 537 million adults living with diabetes globally. The International Diabetes Federation has estimated that diabetes caused around 6.7 million deaths in 2021, and diabetes-related healthcare expenses are increasing, reaching at least USD 966 billion globally. Understanding and addressing T2DM is crucial due to its severe health consequences and substantial economic implications [3].

In the United States, Korean Americans (KAs), a notable minority group estimated at 1.8 million in 2020 [4], exhibit a higher prevalence of T2DM at 10.8% compared to other Asian immigrant groups [5,6]. This minority community faces unique challenges in diabetes management, including language and cultural barriers, limited access to culturally appropriate healthcare, socioeconomic issues, and a general lack of diabetes awareness [7,8]. These obstacles significantly impact their diabetes management and overall quality of life, necessitating a focused approach to improve care outcomes and enhance diabetes-related quality of life in this demographic [9].

Diabetes-specific quality of life (DQOL) is pivotal in understanding the multifaceted experiences of individuals living with T2DM. DQOL extends beyond the general health-related quality of life by focusing on aspects directly influenced by diabetes and its management. This multidimensional construct includes areas such as daily management, satisfaction with treatment, the impact of diabetes, and diabetes-related worry in physical, psychological, and social domains. Physically, it involves diabetes symptoms, daily functioning, and complications. Psychologically, it covers stress, anxiety, depression, and diabetes-related distress. Socially, DQOL reflects relationships, social participation, and experiences of stigma. Previous studies have consistently shown that T2DM can negatively affect these various aspects of quality of life [10,11,12]. Moreover, several studies have found patients with T2D to have a lower QoL than healthy people due to the high demands of treatment, especially if they develop complications, which increase hospitalization, mortality, and disease burden [13,14]. Additionally, minority populations such as KAs might experience unique challenges due to cultural beliefs, language barriers, and different healthcare access patterns, making the study of DQOL in these groups crucial for developing culturally appropriate interventions. 

Lifestyle factors like sleep quality and physical activity significantly affect diabetes management and quality of life. Poor sleep quality, a common issue in individuals with T2DM, can exacerbate symptoms like poor glycemic control and impact overall wellbeing. A recent systematic review has highlighted the negative relationship between sleep quality and quality of life in individuals with T2DM [15]. A cross-sectional study conducted in China also found that poor sleep quality was associated with lower quality of life in patients with T2DM [16]. Another study assessing sleep quality, health-related quality of life, and diabetes-related quality of life reported that poor sleep quality was linked to worse health-related and diabetes-related quality of life in adults with T2DM [17]. Conversely, physical activity can improve quality of life by reducing stress, improving glycemic control, and enhancing general health. Low physical activity can worsen diabetes control, leading to complications and reduced physical health, further impairing quality of life. Several studies have shown that regular physical activity is associated with higher quality of life in individuals with T2DM [18,19]. A systemic review study on the effect of a physical activity intervention program on quality of life in T2DM found that physical activity had a positive effect on health-related quality of life in individuals with the condition [20]. Understanding the role of these lifestyle factors in KAs with T2DM is vital for developing effective, personalized, and culturally tailored diabetes management strategies.

**Figure 1:**
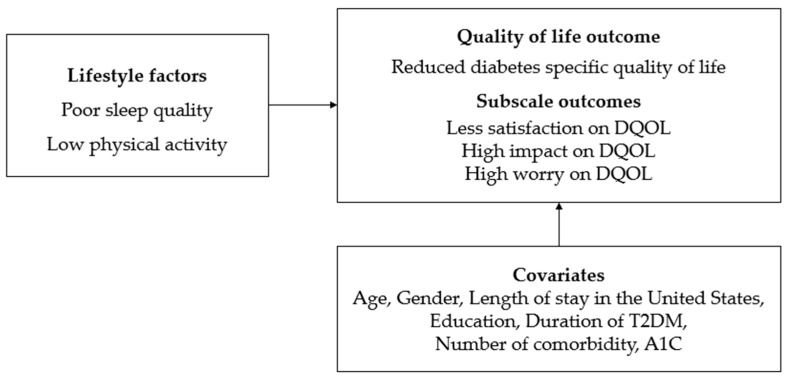
Conceptual framework illustrating the relationships of sleep quality and physical activity with Diabetes Quality of Life (DQOL) among Korean Americans with Type 2 diabetes mellitus (T2DM).

Despite the significant impact of T2DM on KAs and the unique challenges they face, research focusing on the interplay among sleep quality, physical activity, and DQOL in this group is limited. This study aimed to bridge this gap by examining how these lifestyle factors influence DQOL among KAs with T2DM. Therefore, the purpose of this cross-sectional study was to explore the associations between sleep quality and physical activity with DQOL in KAs with T2DM. Figure 1 presents a conceptual framework that systematically illustrates the hypothesized relationships between sleep quality and physical activity with DQOL in KAs diagnosed with T2D. This framework visually delineates how two primary lifestyle factors—sleep quality and physical activity—may influence various aspects of DQOL. It was hypothesized that both poor sleep quality and low levels of physical activity would serve as independent predictors leading to a diminished quality of life, particularly in relation to diabetes management. The outcomes influenced by these lifestyle factors were further categorized into three distinct subscales within the DQOL construct: satisfaction, impact, and worry. The model suggested the following:

- Lower satisfaction with diabetes management was anticipated for participants experiencing poor sleep quality or low physical activity.
- The perceived negative impact of diabetes on daily life was expected to be greater for those with poor sleep quality or low physical activity.
- Increased worry about diabetes was anticipated for those with poor sleep quality or low physical activity.

## 2. Methods

### 2.1. Participants

**Figure 2:**
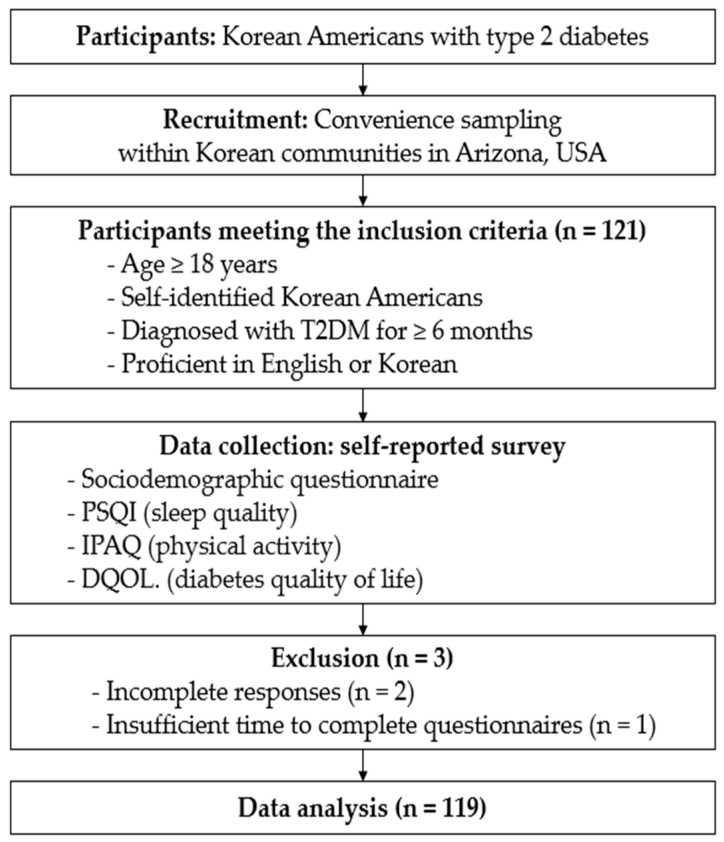
Study flowchart.

As illustrated in the study flowchart in Figure 2, this study utilized convenience sampling to recruit participants from various Korean American communities throughout Arizona. Recruitment locations included churches, a temple, community centers/organizations, Korean markets, and Korean restaurants. The study’s inclusion criteria mandated participants to be as follows: (1) aged 18 years or older, (2) self-identified as Korean Americans, (3) diagnosed with T2DM for at least 6 months prior to data collection, and (4) proficient in reading and writing in English or Korean. The diagnosis of T2DM in this study was based on self-reporting by the participants. The announcements for the study were disseminated in both English and Korean, utilizing study flyers prominently displayed in various settings within the Korean community. The G-power 3.1.9.7 Program was used to determine the required sample size for the study. Considering an effect size of 0.20, an alpha level of 0.05, a power of 80%, 11 predictors, and an anticipated dropout rate of 10%, a minimum sample size of 105 was initially determined. The actual sample size utilized in this study was 119, exceeding the minimum requirement. 

### 2.2. Data Collection

The study, conducted from July to November 2016, involved face-to-face interviews with 121 Korean American adults with T2DM from Arizona, of which 119 completed the necessary questionnaires and measurements. Three participants were excluded due to the following reasons: two participants provided incomplete responses, and one participant had insufficient time to complete the questionnaires. Participants used the Korean versions of the questionnaires. Before data collection, they were informed about the study, its purpose, procedures, benefits, and risks, and provided written informed consent. The survey included self-reported questionnaires on background information, the Pittsburgh Sleep Quality Index (PSQI), the International Physical Activity Questionnaire (IPAQ), and the Diabetes Quality of Life (DQOL) in Korean. After completion, questionnaires were reviewed, and incomplete items were filled in if the participant agreed. The study followed standard ethical procedures for human subject research, ensuring informed consent and data accuracy, and was approved by the Institutional Review Board of the university. 

### 2.3. Measures

The sociodemographic characteristics of the participants were assessed using six key parameters: age, gender, education, length of stay in the US, employment status, and living status. The diabetes-related characteristics were collected through four items on T2DM duration, insulin use, number of comorbidities, and glycosylated hemoglobin (A1C). A1C reflecting average glucose control over the past three months was measured using a finger-stick glucose test by A1C Now. A1C serves as a standard biomarker for glycemic management adequacy. The testing equipment showed a good correlation (*r* = 0.96) with the high-pressure liquid chromatography method [21].

The Pittsburgh Sleep Quality Index (PSQI) is a self-rated questionnaire designed to assess sleep quality and patterns over the past month [22]. It consists of 19 items that cover seven major aspects of sleep: sleep duration, quality, disturbances, latency, daytime dysfunction, habitual sleep efficiency, and use of sleep medication. Each aspect is rated on a scale from 0 to 3, leading to a total possible score ranging from 0 to 21. A global PSQI score greater than 5 suggests clinically significant sleep disturbances, indicating poor sleep quality. The Kaiser–Meyer–Olkin measure of sampling adequacy was confirmed at 0.64, and Bartlett’s test of sphericity supported the factorability of the data with a Chi-Square value of 716.55 and a significance level of *p* < 0.001, demonstrating that the variables are likely correlated in the population. The Korean PSQI version has good internal consistency (Cronbach’s alpha of 0.84) [23]. The reliability of this study yielded a Cronbach’s alpha coefficient of 0.69.

The International Physical Activity Questionnaire-long form (IPAQ) was used in this study to measure the habitual level of physical activity during the past week. The IPAQ is a validated and reliable instrument that has been used in different languages, including English and Korean [24]. The IPAQ consists of 27 items in 6 domains including occupational transport, yard/garden, household, and leisure activities, as well as sitting time. According to the IPAQ guidelines and classification standards [25], physical activity (PA) levels are categorized into 3 groups: low (PA less than 600 min per week), moderate (PA between 600 and 3000 min per week), and high (PA more than 3000 min per week), with higher scores indicating increased physical activity. The Cronbach’s alpha for the IPAQ in this study was 0.70, suggesting it has acceptable internal consistency. 

The Diabetes Quality of Life (DQOL) questionnaire is tailored for individuals with diabetes, consisting of 46 items across three subscales: satisfaction, impact, and worries. The satisfaction subscale, with 15 items, assesses an individual’s satisfaction levels in their life with diabetes, including questions on treatment satisfaction and independence in managing the condition. The impact subscale, comprising 20 items, measures how diabetes affects different life aspects, like work and family life. The worries subscale, containing 11 items, delves into common fears and concerns associated with diabetes, such as worries about future complications and financial burdens. Each item is rated on a five-point Likert scale, where higher scores indicate a poorer diabetes-related quality of life. Developed by the Diabetes Control and Complication Trial Research Group [26], the DQOL has shown good validity in a previous study involving both type 1 and type 2 diabetes, as well as in research specifically with Korean Americans [27,28]. Additionally, the high Kaiser–Meyer–Olkin measure of sampling adequacy (0.92) and the significant Bartlett’s test of sphericity (Chi-Square value of 2631.79, *p* < 0.001) underscore the appropriateness of the sample size and affirm the data’s suitability for the statistical procedure. In the current study, the Cronbach’s alpha values for the total score and each subscale ranged from 0.70 to 0.82.

### 2.4. Analysis

This study utilized a series of statistical analyses to examine the relationship between physical activity and sleep quality with DQOL scores. Descriptive statistics were conducted to characterize the study’s sample in terms of sociodemographic and diabetes-related factors, sleep quality, physical activity, and DQOL and its subscales. For categorical variables, frequencies and percentages were computed, while continuous variables were summarized using means and standard deviations. To evaluate the distribution of continuous variables, the Kolmogorov–Smirnov test was used to assess normality. In the DQOL subscales, the impact and worry variables exhibited non-normal distributions and were thus log-transformed to mitigate this issue. Pearson’s correlations were used to assess the relationships between continuous variables, and Point–Biserial correlations were employed for examining the associations between binary variables such as low and high physical activity and continuous variables. Considering evidence from prior research indicating a strong link with diabetes-related quality of life [17,29,30] and factors that showed significant correlations with DQOL in the current study, this study incorporated gender, age, length of stay in the US, education, T2DM duration, comorbidity, and A1C levels as covariates in the analyses. Two models of multiple linear regression analyses were conducted: Model 1 inspected unadjusted relationships of sleep quality and physical activity with DQOL scores, while Model 2 adjusted for potential confounding variables, including age, gender, education, length of stay in the US, A1C, number of comorbidities, and T2DM duration. Multicollinearity was assessed among the potential predictors—sleep quality, low physical activity, and high physical activity—using Variance Inflation Factor (VIF) and tolerance statistics. The VIF values for sleep quality (1.031 to 1.130), low physical activity (1.172 to 1.374), and high physical activity (1.148 to 1.195) indicated no significant multicollinearity. These results, well below the commonly used threshold of 5, confirm the statistical independence of our predictors and validate the integrity of our regression analysis. This multicollinearity assessment ensures the findings on the DQOL and its subscales are based on a robust statistical foundation. The reliability and validity of the measurements in this study were evaluated using Cronbach’s alpha coefficients for reliability assessment and factor analysis for validity assessment. All analyses were performed using SPSS version 27.0, with statistical significance set at *p* < 0.05.

## 3. Results

### 3.1. Descriptive Analyses of the Participants

A total of 119 participants, including 82 women and 37 men, completed all survey items. Table 1 provides a detailed account of the sociodemographic and diabetes-related characteristics of these participants. The participants had an average age of 67.0 years, with a standard deviation of 9.68 years, and their age range was 43 to 85 years. On average, participants had been residing in the United States for over 33 years, with a standard deviation of 10.45 years. More than half of the participants had educational qualifications of high school graduation or lower. About 34% of the participants were employed, while a majority, 70.6%, were living with a partner.

Regarding diabetes-related characteristics, the mean duration of T2DM among participants was 9.84 years, with a standard deviation of 9.65 years. Only 10 participants (8.4%) were using insulin. Over a quarter of the participants had three or more comorbidities, 37% had 2 comorbidities, and 14.3% had no comorbidity. The average glycosylated hemoglobin (A1C) level was 7.0%, with a standard deviation of 1.09%. 

As can be found in Table 2, participants reported a mean sleep quality score of 7.55 ± 3.67, and the majority (66.4%) had poor sleep quality with PSQI of more than 5 scores. Over a third (33.6%) of participants showed low physical activity, and the median of physical activity was 15 (IQR = 6.83–38.80) hours per week. The mean score of the total DQOL was 1.85 (SD = 0.28), with a range from 1.28 to 2.83. In the 3 subscales of DQOL, the mean scores of satisfaction, impact, and worry were 2.80 (SD = 0.47), 1.51 (SD = 0.39), and 1.16 (SD = 0.22), respectively.

### 3.2. Correlations of Sleep Quality and Physical Activity on Diabetes Quality of Life and Subscales

Table 3 shows bivariate correlations of sleep quality and physical Activity on DQOL and subscales. Sleep quality was positively related to DQOL, satisfaction, and impact (*r* = 0.32, *r* = 0.20, *r* = 0.34, respectively). Low physical activity had a moderately positive correlation with DQOL, satisfaction, and impact (*r*= 0.34, *r*= 0.29, *r*= 0.30, respectively), whereas high physical activity showed significantly negative relationships with DQOL and impact (*r* = −0.24 and *r* = −0.25, respectively).

### 3.3. Relationships of Sleep Quality and Physical Activity with Diabetes Quality of Life and Subscales

Table 4 describes the unadjusted and adjusted multiple linear regression analyses modeling the associations of sleep quality and physical activity with the total score and subscales of DQOL. In the total of DQOL, sleep quality (*β* = 0.26, *p* = 0.002 and *β* = 0.24, *p* = 0.004, respectively) and low level of physical activity (*β* = 0.25, *p* = 0.007 and *β* = 0.18, *p* = 0.043, respectively) were independent predictors of DQOL in both unadjusted and adjusted models. In the subscales of DQOL, satisfaction had a significant association with only low physical activity, regardless of models (*β* = 0.25, *p* = 0.011 and *β* = 0.22, *p* = 0.023, respectively), suggesting that enhancing physical activity levels could potentially boost satisfaction within DQOL. Interestingly, the impact was positively related to sleep quality (*β* = 0.29, *p* < 0.001) and low physical activity (*β* = 0.19, *p* = 0.034) in the unadjusted model. However, only sleep quality (*β* = 0.25, *p* = 0.004) and not low activity remained a significant predictor of impact after adjustment for covariates, indicating that better sleep quality might significantly lower the perceived negative impact of diabetes. No significant associations of sleep quality and physical activity with worry were found in either unadjusted or multivariate-adjusted models. 

## 4. Discussion

This study explored the associations of sleep quality and physical activity with Diabetes Quality of Life (DQOL) among Korean Americans (KAs) with type 2 diabetes (T2DM). The study employed a validated instrument for the self-reported assessment of DQOL, revealing that participants, on average, experienced a moderate level of diabetes-related quality of life. Notably, the overall mean DQOL score obtained in the current study was somewhat lower than the mean scores documented in earlier research utilizing the same DQOL measure [5,17]. This suggests that participants in the current investigation reported a marginally improved quality of life in relation to their diabetes management and experiences compared to individuals in prior studies. One plausible explanation for this difference may include advancements in diabetes care and management strategies, which could contribute to enhanced daily living and reduced diabetes-related distress among participants. In terms of the subscales of DQOL, the study found that participants reported relatively worse diabetes life satisfaction on average, a moderate level of impact of diabetes, and a low level of worry related to their diabetes. These findings provide insight into the specific areas of diabetes-related quality of life that may be particularly important to address in interventions aimed at improving overall quality of life among KAs with T2DM. A previous study showed the highest mean score in the subscales of the DQOL was satisfaction [17], which was consistent with the current study. This study indicates that individuals with T2DM may be less satisfied with their lives than with the impact of diabetes or the worry about diabetes. The possible reason is that people with T2D are more likely to have a poor quality of life due to the challenges and limitations that come with managing the condition, such as dietary restrictions, medication regimens, and regular monitoring of blood glucose levels. Additionally, the stress associated with diabetes management can also influence overall life satisfaction [31,32,33]. Thus, this present study suggests that tailored interventions aimed at improving overall quality of life among individuals with T2DM may need to address specific areas related to diabetes life satisfaction, such as medical treatment or diabetes self-management.

The findings of this study revealed a significant correlation: individuals with poor sleep quality and lower levels of physical activity reported a reduced quality of life in relation to their diabetes management. Additionally, approximately 70% of participants suffered from poor sleep quality, and a third exhibited low levels of physical activity. The primary findings indicate that sleep quality and physical activity independently predict DQOL in KAs with T2DM. This pattern is consistent with findings from various racial and ethnic groups. A systematic review examined the link between sleep quality and quality of life in T2DM, including diverse racial groups such as Americans, Indians, Chinese, Japanese, Canadians, Spaniards, Italians, and Brazilians. The review found that improving sleep quality can have both direct and indirect positive effects on the quality of life among this population [15]. Another systematic review assessed the effect of physical activity interventions on the quality of life in people with T2DM and found that aerobic exercise had a significant positive effect compared to the control group [20]. A cross-sectional study in the USA found that poor sleep quality was associated with decreased health-related and diabetes-related quality of life in adults with T2DM [17]. Furthermore, a cross-sectional study conducted among rural Chinese elderly found that physical activity improved the quality of life and moderated the relationship between sleep quality and quality of life [34]. These collective findings emphasize the vital roles of sleep quality and physical activity in managing the quality of life for people with T2DM, underscoring their importance across diverse cultural and racial backgrounds. 

In the subscales of DQOL, participants with lower levels of physical activity reported reduced satisfaction in their lives with diabetes. The finding indicates a clear link between lower levels of physical activity and less satisfaction with life among KAs with T2DM, suggesting that interventions aimed at increasing physical activity could have a pronounced positive effect on the satisfaction component of DQOL. Furthermore, the impact subscale, which assesses the perceived negative influence of diabetes on daily life, showed that those experiencing worse sleep quality had a greater negative impact on their diabetes management. This highlights the pivotal role of sleep quality in mitigating the perceived adverse effects of diabetes on an individual’s life, underscoring the importance of addressing sleep issues as part of comprehensive diabetes management. Contrastingly, the findings of the current study did not reveal any significant associations between either sleep quality or physical activity and the worry subscale of DQOL. This absence of correlation might be attributed to the design of the worry subscale, which may resonate more with younger diabetes patients, considering the age of the participants in this study. These findings are consistent with previous studies showing that adults who had low levels of physical activity were more likely to be less satisfied with their quality of life compared to those who were physically active [35,36]. A previous study using the same DQOL instrument reported that satisfaction and impact subscales were significant predictors of sleep quality [17]. Another study reported that sleep quality indirectly affects the relationship between psychological distress and DQOL [37]. An intervention study also reported that sleep quality indirectly influenced quality of life via physical, mental, and social well-being [38]. Furthermore, poor sleep quality can worsen diabetes symptoms by impacting glucose metabolism, appetite regulation, and stress hormones. In turn, this can adversely affect family and work life, leading to a reduced quality of life. Similarly, low physical activity is a well-known risk factor for poor glycemic control and can lead to complications in diabetes management, which can lead to less satisfaction with living with diabetes. To expand upon these findings, it is crucial to consider the broader implications for diabetes care and management. The significant associations observed between lifestyle factors and specific DQOL subscales underscore the potential benefits of holistic approaches to diabetes management that incorporate strategies for improving sleep quality and increasing physical activity. Such approaches could not only enhance overall quality of life but also address key areas of concern, such as life satisfaction and the perceived impact of diabetes. Future research should explore targeted interventions that address these lifestyle factors, potentially offering valuable strategies for improving the well-being of KAs living with T2DM.

The findings contribute to existing knowledge by providing empirical evidence from a demographic that is underrepresented in diabetes research. The study highlights the importance of considering ethnic variations in diabetes care and management. It challenges the traditional focus on glycemic control through medication and diet and emphasizes the role of lifestyle factors like sleep and physical activity. This study suggests that these areas should be integral components of diabetes management programs. Additionally, the study raises questions about the potential unique stressors and lifestyle factors faced by KAs that may influence their sleep quality and physical activity levels, thus affecting diabetes management. This calls for further research into culturally specific factors that impact diabetes care. In summary, this study corroborates existing findings on the importance of sleep and physical activity in improving Diabetes Quality of Life while providing new insights specific to the Korean American population. It emphasizes the need for a holistic, culturally sensitive approach to diabetes care to improve the quality of life for individuals managing this chronic condition.

The findings of this study have significant implications for clinical practice, especially in the context of caring for KAs with T2DM. Healthcare professionals working with KAs with T2DM should prioritize regular assessments of sleep quality and physical activity as essential components of diabetes management. Clinicians can use validated tools to evaluate sleep patterns and customize physical activity recommendations based on individual patient needs, while considering cultural preferences and lifestyle. The findings from this study also have considerable implications for local public health practice in Arizona, particularly in the design and implementation of diabetes management programs targeting the Korean American population. Public health officials and community health organizations should consider incorporating sleep quality improvement and physical activity promotion into their diabetes care strategies. By offering culturally tailored education sessions, community-based physical activity programs, and resources for improving sleep hygiene, these initiatives can address the specific needs and preferences of KAs with T2DM. Engaging community leaders and leveraging social networks within Korean communities can further enhance the reach and effectiveness of such programs, ultimately leading to improved diabetes outcomes and quality of life for this population.

This study has several limitations. The cross-sectional design does not allow for inferring causality between the variables examined. Longitudinal studies are needed to better understand the temporal relationship between diabetes-related factors and quality of life. The convenience sample limited to KAs with T2DM living in Arizona, USA, may limit the generalizability of the findings. Future studies with more diverse populations could provide a more comprehensive understanding of the relationship between diabetes-related factors and quality of life. Additionally, this study did not consider other potential factors known to influence DQOL, which could have affected the results [39,40]. Future studies should include these variables in their analyses to gain a more comprehensive understanding of the factors contributing to DQOL in KAs with T2DM. Lastly, it would be beneficial for future research to extend upon our findings by considering the effects of the post-COVID-19 era, as well as the role of digital health technologies and social networks in managing diabetes and improving quality of life. 

The roadmap for future research on the quality of life (DQOL) among Korean Americans with type 2 diabetes mellitus (T2DM) encompasses several strategic priorities. To gain a clearer understanding of the causal relationships between lifestyle factors—such as sleep quality and physical activity—and DQOL, future studies should prioritize longitudinal designs. Broadening the scope of participant demographics beyond the initial convenience sample from Arizona to include a wider range of demographic and geographic backgrounds will enhance the generalizability of the findings. Additionally, integrating variables that have been previously overlooked but are known to influence DQOL will enrich our understanding of its determinants. In the wake of data collection for this study, significant time was devoted to the processes of data analysis, manuscript preparation, and publication. Given this, future research should also aim to examine the impact of the COVID-19 pandemic on diabetes management and DQOL. This includes addressing both the challenges and opportunities that have emerged, such as changes in healthcare delivery, patient behavior, and access to services. Investigating the post-COVID-19 era’s effects on diabetes management and quality of life, alongside the potential role of digital health technologies and social networks, offers a promising path for exploring how these factors can be seamlessly integrated into effective diabetes care strategies. Adopting this comprehensive approach will not only build upon the findings of the current study but also foster a more holistic understanding of diabetes management that emphasizes patient well-being and quality of life.

## 5. Conclusions

This study highlights the significant roles of sleep quality and physical activity as independent predictors of Diabetes Quality of Life (DQOL) among Korean Americans (KAs) with type 2 diabetes mellitus (T2DM). In particular, the findings revealed that poor sleep quality and lower physical activity levels were associated with reduced life satisfaction and a greater perceived negative impact of diabetes, without significantly affecting diabetes-related worry. These results underscore the critical need to integrate lifestyle factors into diabetes management strategies, advocating for a comprehensive approach that extends beyond conventional medication and diet modifications to include personalized interventions. 

For clinical practice, the study suggests prioritizing assessments of sleep quality and physical activity in the care of KAs with T2DM. Tailoring interventions to individual needs and cultural preferences can enhance diabetes outcomes. Public health strategies should also include sleep improvement and physical activity promotion, particularly within individually tailored programs for the Korean American community. Ultimately, these approaches seek to improve the quality of life for those living with diabetes by minimizing adverse effects and boosting satisfaction.

## Figures and Tables

**Table 1:** Sociodemographic and diabetes-related characteristics of the participants.

Variables	Frequency (%)	Mean ± SD	Actual Range
Gender			
Men	37 (31.1)		
Women	82 (68.9)		
Age (years)		67.00 ± 9.68	43–85
Length of stay in the US (years)		33.72 ± 10.45	7–54
Education			
≤High school	62 (52.1)		
>High school	57 (47.9)		
Employment			
Employed	40 (33.6)		
Unemployed	79 (66.4)		
Living			
With partner	84 (70.6)		
Without partner	35 (29.4)		
Duration of T2DM (years)		9.84 ± 9.65	1–42
Use of insulin			
Yes	10 (8.4)		
No	109 (91.6)		
Number of comorbidities			
0	17 (14.3)		
1	28 (23.5)		
2	44 (37.0)		
3 or more	30 (25.2)		
A1C (%)		7.00 ± 1.09	5.4–10.4

Note. A1C = glycosylated hemoglobin; SD = standard deviation.

**Table 2:** Descriptive statistics of the study variables (*N* = 119).

Variables	Frequency (%)	Mean ± SD	Actual Range
Sleep quality (PSQI)		7.55 ± 3.67	1–17
Poor sleep (PSQI > 5)	79 (66.4)		
Good sleep (PSQI ≤ 5)	40 (33.6)		
Physical activity (IPAQ: hour/week), median (IQR)		15.00 (6.83–38.80)	0–104.70
Low activity	40 (33.6)		
Moderate activity	55 (46.2)		
High activity	24 (20.2)		
Diabetes Quality of Life: DQOL		1.85 ± 0.28	1.28–2.83
Satisfaction		2.80 ± 0.47	1.33–3.80
Impact		1.51 ± 0.39	1.00–3.30
Worry		1.16 ± 0.22	1.00–2.36

Note. IPAQ = International Physical Activity Questionnaire; PSQI = Pittsburgh Sleep Quality Index; DQOL = Diabetes Quality of Life; SD = standard deviation; IQR = interquartile range.

**Table 3:** Bivariate correlations of sleep quality and physical activity on DQOL and subscales (*N* = 119).

Variables	DQOL	Subscales of DQOL		
		Satisfaction	Impact	Worry
Sleep quality	0.32 ***	0.20 *	0.34 ***	0.01
Low physical activity	0.34 **	0.29 **	0.30 ***	0.06
High physical activity	−0.24 **	−0.15	−0.25 **	−0.06

Note. DQOL = Diabetes Quality of Life. * *p* < 0.05; ** *p* ≤ 0.01; *** *p* ≤ 0.001.

**Table 4:** Multiple linear regression models of Diabetes Quality of Life and subscales (*N* = 119).

	DQOL						Subscales of DQOL					
				Satisfaction			Impact			Worry		
	*β*	*S.E.*	*p*	*β*	*S.E.*	*p*	*β*	*S.E.*	*p*	*β*	*S.E.*	*p*
Model 1												
Sleep quality	0.26	0.006	0.002	0.15	0.011	0.097	0.29	0.001	0.000	−0.01	0.001	0.943
Physical activity												
Low activity	0.25	0.053	0.007	0.25	0.094	0.011	0.19	0.012	0.034	0.05	0.009	0.612
Moderate activity	1			1			1			1		
High activity	−0.12	0.062	0.170	−0.05	0.110	0.589	−0.15	0.014	0.090	−0.04	0.010	0.691
Model 2												
Sleep quality	0.24	0.006	0.004	0.14	0.011	0.112	0.25	0.001	0.004	0.05	0.001	0.562
Physical activity												
Low activity	0.18	0.052	0.043	0.22	0.092	0.023	0.13	0.012	0.174	−0.01	0.008	0.913
Moderate activity	1			1			1			1		
High activity	−0.10	0.057	0.242	−0.04	0.100	0.670	−0.13	0.013	0.157	−0.02	0.009	0.801

Note. Model 1: unadjusted, Model 2: adjustment for gender, age, length of stay in the United States, education, duration of T2DM, number of comorbidities, and A1C. *β* = beta; *S.E.* = standard error; DQOL = Diabetes Quality of Life.

## Data Availability

The data supporting the findings of this study are available from the author upon reasonable request.

## Abbreviations

T2DM	Type 2 Diabetes Mellitus
DQOL	Diabetes Quality of Life
Kas	Korean Americans
PSQI	Pittsburgh Sleep Quality Index
IPAQ	International Physical Activity Questionnaire
SD	Standard Deviation
A1C	Glycosylated Hemoglobin
IQR	InterQuartile Range
